# Memristive True Random Number Generator for Security Applications

**DOI:** 10.3390/s24155001

**Published:** 2024-08-02

**Authors:** Xianyue Zhao, Li-Wei Chen, Kefeng Li, Heidemarie Schmidt, Ilia Polian, Nan Du

**Affiliations:** 1 Institute for Solid State Physics, Friedrich Schiller University Jena, 07743 Jena, Germany; xianyue.zhao@uni-jena.de (X.Z.); kefeng.li@uni-jena.de (K.L.); heidemarie.schmidt@uni-jena.de (H.S.); 2 Department of Quantum Detection, Leibniz Institute of Photonic Technology (IPHT), 07745 Jena, Germany; 3 Institute of Computer Science and Computer Engineering, University of Stuttgart, 70569 Stuttgart, Germany; li-wei.chen@informatik.uni-stuttgart.de (L.-W.C.); ilia.polian@informatik.uni-stuttgart.de (I.P.)

**Keywords:** TRNG, hardware security, entropy source, digital memristor, YMO memristor

## Abstract

This study explores memristor-based true random number generators (TRNGs) through their evolution and optimization, stemming from the concept of memristors first introduced by Leon Chua in 1971 and realized in 2008. We will consider memristor TRNGs coming from various entropy sources for producing high-quality random numbers. However, we must take into account both their strengths and weaknesses. The comparison with CMOS-based TRNGs will serve as an illustration that memristor TRNGs stand out due to their simpler circuits and lower power consumption— thus leading us into a case study involving electroless YMnO_3_ (YMO) memristors as TRNG entropy sources that demonstrate good security properties by being able to produce unpredictable random numbers effectively. The end of our analysis sees us pinpointing challenges: post-processing algorithm optimization coupled with ensuring reliability over time for memristor-based TRNGs aimed at next-generation security applications.

## 1. Introduction

The concept of the memristor was first theorized in 1971 by Leon Chua [1], who postulated it as the fourth fundamental circuit element alongside the resistor, capacitor, and inductor. However, it was not until 2008 that a practical realization of the memristor was achieved by researchers at HP Labs [2], who demonstrated a titanium-dioxide-based device that exhibited memristive behavior. Since then, extensive research has been conducted to explore different materials and structures to optimize the performance of memristors for specific applications [3,4,5,6,7].

One of the most compelling applications of memristive technology is in the field of hardware security, particularly in the development of true random number generators (TRNGs) [8,9,10,11,12,13]. TRNGs are critical components in cryptographic systems, providing the unpredictability required for secure encryption keys and other security protocols. Memristors, with their inherent stochastic switching properties, offer a novel approach to generating high-quality random numbers.

The first introduction of a memristor-based TRNG in 2012 marked a pivotal moment for security applications using memristive technology [8]. Initial designs using W/TiO_*x*_N_*y*_/SiO_2_ structures exploited random telegraph noise (RTN) as an entropy source for TRNGs, although they faced challenges with activation and voltage control. Later research explored Pt/TaO_*x*_/Ta memristors, focusing on cycle-to-cycle (C2C) variations as an entropy source for TRNGs [11]. Despite improvements, these designs have not fully passed National Institute of Standard and Technology (NIST) tests without post-processing [8,9,11]. The Pt/Ag/Ag:SiO_2_/Pt memristor-based TRNG achieves endurance of $10^{7}$ cycles and a bit generation rate of 6 kb/s using delay time as the entropy source. This development is promising as it passes all 15 NIST tests, demonstrating that high randomness quality can be achieved without post-processing [14]. The NIST test, which consists of a total of 15 tests, is recognized as the most important for analyzing the long binary sequences generated by various TRNGs to evaluate the randomness of the TRNG. Passing all of these tests indicates a high level of statistical randomness, meaning that the generated bits appear random and unbiased. However, it should be noted that passing these tests is necessary but not sufficient to ensure the overall security and reliability of the TRNG. Other factors must also be considered, such as resistance to physical attack and long-term stability.

In recent years, research on memristor-based TRNGs has grown rapidly. However, the review literature on memristor-based TRNGs presents memristor-based TRNGs as a branch of memristor-based hardware security, which is not systematic and complete [15,16,17,18,19]. Our review and analysis of recent research on memristors in TRNGs can fill this gap. This work provides a comprehensive overview of the current state of memristor-based TRNGs, highlighting the advances and challenges in the field. Our review covers almost all important research, from the earliest use of memristors as entropy sources in TRNGs to the most recently published studies. In a comparative discussion with CMOS-based TRNGs, the scenarios and possibilities of future applications of memristor-based TRNGs are presented. Furthermore, a case study presents an approach using an electroforming-free YMnO_3_ (YMO) memristor as the core component of a TRNG. This digital memristor is well suited as an entropy source for a TRNG. A YMO memristor-based TRNG can achieve high randomness without much post-processing.

## 2. Memristive Technology and Its Application Scenarios

Memristive technology is attracting attention as it has the potential to completely alter our understanding of computations and storage [20]. As the most basic characteristic of a memristor, it can maintain its resistive state before change, providing an alternative to conventional storage systems as a high-density, low-power nonvolatile memory [21]. The integration of memristors into modern technology promises advancements in neuromorphic computing, which mimics neural architectures for more efficient and adaptive computation [22,23,24]. In arithmetic computing applications, a memristor acts both as a site for storing data and logic computations [25,26]. Additionally, their application extends to the creation of physical unclonable functions (PUFs) and TRNGs for enhanced hardware security [12,15,27]. These rich application scenarios illustrate the versatility of memristive devices.

**Figure 1:**
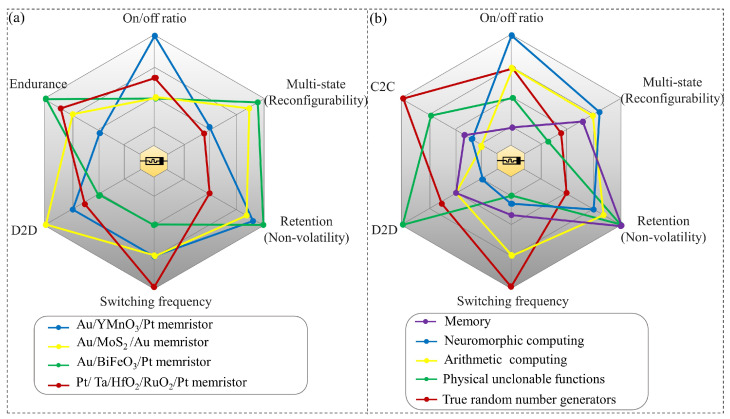
(**a**) Comparative analysis of various memristor types and their key performance parameters: on/off ratio, multistate (reconfigurability), retention (nonvolatility), switching frequency, D2D variation, and endurance. (**b**) Application scenarios of memristive technology and their key performance parameters: on/off ratio, multistate (reconfigurability), retention (nonvolatility), switching frequency, D2D variation and C2C variation.

Figure 1 comprehensively compares various memristive materials and their performances, alongside the potential application scenarios these performances enable. The radar charts visually represent key performance such as the on/off ratio, endurance, C2C variation, device-to-device (D2D) variation, switching frequency, retention (nonvolatility), and multistate (reconfigurability). These metrics are crucial in determining the suitability of memristive materials for specific applications.

Figure 1a illustrates the performance characteristics of four distinct memristive materials: Au/YMnO_3_/Pt [28], Au/MoS_2_/Au [29], Au/BiFeO_3_/Pt [30], and Pt/Ta/HfO_2_/RuO_2_/Pt [31]. Each material is evaluated across six critical parameters: on/off ratio, multistate (reconfigurability), retention (nonvolatility), switching frequency, D2D variation, and endurance. The Au/YMnO_3_/Pt memristor exhibits superior performance in on/off ratio and switching frequency, while the Au/BiFeO_3_/Pt memristor excels in endurance and multistate capabilities. The Au/MoS_2_/Au memristor shows balanced performance across all metrics, with a slight advantage in retention. The Pt/Ta/HfO_2_/RuO_2_/Pt memristor demonstrates the highest D2D performance but lags in switching frequency.

Comparing these memristors, we observe trade-offs between different performance aspects. The Au/YMnO_3_/Pt memristor’s high on/off ratio and switching frequency make it suitable for applications requiring fast, distinct state changes. The Au/BiFeO_3_/Pt memristor’s strong endurance and multistate capabilities suggest its potential for complex, long-lasting computational tasks. The Au/MoS_2_/Au memristor’s balanced profile indicates versatility across various applications. The Pt/Ta/HfO_2_/RuO_2_/Pt memristor’s high D2D performance implies consistency in manufacturing, which is crucial for large-scale integration, despite its lower switching frequency.

Figure 1b correlates the parameters with five application scenarios: memory storage, neuromorphic computing, arithmetic computing, PUFs, and TRNGs. The radar chart evaluates these applications based on the same five performance metrics as in Figure 1a, with the addition of C2C. Memory storage applications show moderate requirements across all metrics, indicating the need for reliability and versatility in data storage. Neuromorphic computing requires high performance in on/off ratio and multistate capabilities, reflecting the need for precise, multilevel synaptic weights in artificial neural networks. Arithmetic computing requires balanced performance, with particular emphasis on switching frequency for fast computation. PUFs prioritize D2D variation and retention, critical for maintaining unique, stable security keys. TRNGs have a distinct profile with high requirements for switching frequency and C2C variation, essential for generating unpredictable sequences.

### 2.1. Classifications of Memristors

As a nonlinear two-terminal electrical component, the conductivity of a memristor can be tuned by adjusting the magnitude, direction, or duration of its terminal voltage [32,33]. The most representative characteristic of the memristor is its I–V curve, which exhibits a pinched hysteresis loop [34]. Based on the shape of the I–V curve, they can be roughly classified as digital memristor and analog memristor. The resistance of digital memristors shows abrupt changes at higher resistance ratios, making them ideal for use in memory and arithmetic computing. Analog memristors exhibit gradual changes in resistance and are therefore more suitable for analog circuits and neuromorphic computing. In addition, in recent years, some memristors have been classified as dual-mode memristors, offering resistive characteristics and digital/analog dual-mode operability [31,35,36]. This opens up more possibilities for the application of memristors.

Digital memristors, such as the Au/YMnO_3_/Pt and Au/MoS_2_/Au devices, are characterized by their ability to abruptly change between different discrete resistive states [28,29]. These memristors are ideal for applications that require precise and abrupt switching between two states, such as in nonvolatile memory storage and digital logic operations. The YMO memristors, for instance, have shown electroforming-free unipolar resistive switching with high voltages and currents required for the SET ($V_{\mathrm{SET}}$) and RESET ($V_{\mathrm{RESET}}$) processes. Their endurance is in the order of $10^{3}$ for specific compositions. In terms of retention, these memristors have demonstrated stable properties, maintaining a large memory window characterized by the OFF to ON resistance ratio in the order of $10^{4}$–$10^{6}$. This large memory window is advantageous for applications in memory and logic devices, as it allows for a clear distinction between the HRS and LRS.

Analog memristors, such as the Au/BiFeO_3_/Pt device, are designed to exhibit a continuum of resistance levels, facilitating a more nuanced representation of information [30]. This characteristic makes them suitable for neuromorphic computing and analog signal processing. The BiFeO_3_ memristors have shown remarkable endurance, maintaining stable LRS and HRS with $3\times 10^{4}$ cycles. Additionally, these memristors can retain stable states for over a decade at various temperatures, indicating their suitability for low power consumption and persistent data storage applications. The on/off ratio in these memristors is approximately $10^{2}$, which is crucial for precise data storage and retrieval.

The Ta/HfO_2_/RuO_2_ stacked memristor is an example of a dual-mode device, capable of operating in both digital and analog modes [31]. This functionality is enabled by the rupture and rejuvenation of the conductive filament and its geometric variation during a multiphase reset process. In the analog mode, achieved after a gradual reset, the device can define 256 stable states. In contrast, the digital mode, achieved after a full reset, provides discrete resistive switching between low and high resistance states with minimal variation. The Ta/HfO_2_/RuO_2_ memristors have shown an exceptional endurance exceeding $10^{5}$ switching cycles for both modes, with each state exhibiting high retention up to $10^{4}$ s at 85°C without degradation. A large memory window of two orders of magnitude, including C2C variation, confirms their ability to provide a distinct ON/OFF ratio of up to $10^{3}$. This dual-mode functionality, combined with low variation and precise conductance tunability, positions the Ta/HfO_2_/RuO_2_ memristor as a state-of-the-art device for mixed-signal processors in neuromorphic applications.

The classifications of memristors highlight their diverse applications and operational capabilities. Digital memristors are preferred for applications requiring binary switching, while analog memristors are more suitable for applications that benefit from a continuous range of resistance states. Dual-mode memristors, such as the Ta/HfO_2_/RuO_2_ device, offer the flexibility of both digital and analog operations, making them highly versatile for a range of applications, including nonvolatile memory, logic operations, neuromorphic computing, and mixed-signal processing. The endurance, retention, and ON/OFF ratio of these memristors are critical parameters that influence their suitability for specific applications.

### 2.2. Key Performance Parameters

Here, we discuss seven common parameters that determine the performance of memristors: on/off ratio, multistate capability (reconfigurability), retention (nonvolatility), switching frequency, D2D variation, C2C variation, and endurance. Each of these parameters plays a crucial role, as it helps to indicate that memristors would be preferred for different applications—from neuromorphic computing to high-density storage systems—based on their suitability.

The on/off ratio, a fundamental characteristic of memristors, quantifies the resistance contrast between the device’s high and low resistance states. This parameter is crucial for ensuring reliable state differentiation, and directly impacts the device’s ability to store and process information accurately. A high on/off ratio facilitates a clear distinction between logical states, enhancing the robustness of computations and storage operations in memristive systems.

Multistate capability, or reconfigurability, refers to a memristor’s ability to exhibit multiple stable resistance states. This property is particularly valuable for implementing analog computing paradigms and mimicking synaptic plasticity in neuromorphic architectures. The degree of reconfigurability determines the granularity of information that can be encoded within a single device, potentially enabling more efficient and compact computational systems.

Retention, also known as nonvolatility, describes a memristor’s ability to maintain its resistance state without external power. This characteristic is essential for persistent memory applications and energy-efficient computing paradigms. High retention capabilities ensure data integrity over extended periods, reducing the need for frequent refresh operations and minimizing power consumption in standby modes.

Switching frequency denotes the speed at which a memristor can transition between resistance states. This parameter is critical for applications requiring high-speed operations, such as real-time processing and high-bandwidth memory systems. The switching frequency often involves a trade-off with other parameters, such as endurance and energy efficiency, necessitating careful optimization for specific use cases.

Device-to-device variation quantifies the consistency of electrical characteristics across different memristive devices. Minimizing D2D variation is crucial for ensuring reliable operation in large-scale integrated circuits and systems. High D2D variation can lead to unpredictable behavior and reduced yield in manufacturing processes, posing challenges for the widespread adoption of memristive technologies.

Cycle-to-cycle variation refers to the consistency of a memristor’s behavior over repeated switching operations. This parameter is particularly important for applications requiring long-term stability and predictability, such as in-memory arrays or neuromorphic learning systems. Minimizing C2C variation ensures consistent performance over the device’s lifetime and enhances the reliability of memristive circuits.

Endurance, the final key parameter, measures a memristor’s ability to withstand repeated switching operations without significant degradation in performance. High endurance is crucial for applications involving intensive read/write cycles, such as in-memory computing or frequently accessed storage systems. The endurance of memristive devices directly impacts their long-term reliability and suitability for demanding computational tasks.

### 2.3. Application Scenarios

Memristive devices emerge as compelling candidates, offering nonvolatile memory elements characterized by high density, fast access, and low power consumption. These attributes position memristive memory as a viable alternative to traditional memory technologies. Figure 1b illustrates the broad application scenarios of memristive technology, encompassing memory, neuromorphic computing, arithmetic computing, PUFs, and TRNGs.

The analog nature of memristive devices lends itself well to neuromorphic computing, enabling the efficient implementation of artificial neural networks and spiking neural networks. This capability opens avenues for brain-inspired computing, with memristive devices serving as key components in neuromorphic architectures. Arithmetic computing benefits from memristive crossbar arrays, which excel in performing matrix-vector multiplication in-memory. This capability accelerates arithmetic-intensive workloads, offering potential efficiency gains in various computational tasks. PUFs leverage the inherent variability of memristive devices to generate unique device fingerprints for hardware security applications. This feature enhances device authentication and security, bolstering protection against unauthorized access and tampering. TRNGs capitalize on the stochastic switching of memristive devices to generate truly random numbers, crucial for cryptographic applications and secure communications. Memristive TRNGs offer inherent randomness, contributing to robust encryption and data security. These applications leverage the unique characteristics of memristive devices to enable novel computing paradigms with potential implications for various fields.

Despite significant advancements in memristor technology, the quest for a perfect memristor with ideal retention and endurance characteristics remains ongoing. Various memristor types exhibit unique advantages and limitations, highlighting the need for tailored solutions for specific applications. Furthermore, there are memristors with ultra-short switching time (high speed to 120 ps [37]), ultra-high density [38], excellent retention (greater than 10 years [39,40]), and the best-known endurance (more than $10^{12}$ [41]), but there is no single memristor technologies that could have all the features mentioned above. Nevertheless, researchers navigate these trade-offs by leveraging the strengths of different memristor technologies to optimize performance for diverse application scenarios. Ultimately, addressing retention and endurance challenges in memristors requires interdisciplinary approaches integrating materials science, device physics, and circuit design to unlock their full potential in emerging computing paradigms and memory architectures.

The central application of memristors in the context of this study is the generation of true random numbers. Modern TRNGs include a source of randomness, which can be memristive, but they also incorporate modules for post-processing, buffering, and online self-test. A TRNG for use in cryptographic applications must, in addition to good statistical properties, also maintain forward and backward secrecy, i.e., make it intractable for an attacker who gained access to one or several values produced by the TRNG to derive its future or previous outputs. Specifically, the German AIS-31 standard [42] demands the formulation of a stochastic model, which describes the randomness source and allows the calculation of a lower bound for the entropy of the produced bit stream (this model should not be confused with stochastic models of memristive devices, e.g., [43]). It should take into account the influence of environmental parameters, such as temperature or operating voltage, and aging. Online tests must make sure that the parameters under which the TRNG is operated are within the range for which the stochastic model has been derived.

While a complete TRNG system must be considered for its certification and practical use, this article focuses on the memristive randomness source. Post-processing circuitry (memristive or not), test procedures, and stochastic model of the integrated TRNG are considered as future work.

## 3. True Random Number Generator Based on Memristors

Unpredictability in applications hinges on the deployment of random bit generators, which derive their entropy from nondeterministic phenomena. In integrated circuit implementations, electronic noises (thermal and shot) and time jitter serve as the primary entropy sources [44].

**Figure 2:**
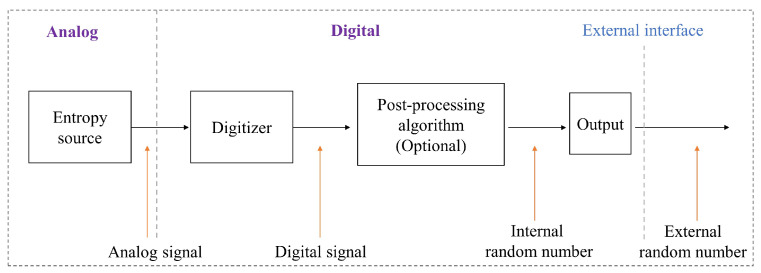
Generic design of a TRNG.

Figure 2 depicts the architecture of a TRNG, illustrating its fundamental components, where the definitions reported in [44,45] have been adopted. The entropy source produces an analog signal that is subsequently processed by a digitizer. This digitizer samples the analog signal and transforms the sampled values into random bits.

To enhance the statistical quality of these bits, they may undergo algorithmic post-processing. This processed bit stream is then directed into a post-processing unit, which outputs a sequence of random words, referred to as internal random numbers. The post-processing unit fulfills two critical roles. Firstly, it adjusts the probability distribution of the raw random bits to mitigate any statistical deficiencies arising from the nondeterministic noise sources or the digitizer, such as input offsets in voltage comparators. As a common method, the raw bit stream first goes through von Neumann correction before being tested by NIST, a common post-processing method used to remove “1” and “0” deviations from the sequence. This adjustment yields a more uniform probability distribution for the resulting random bit stream compared to the input raw bit stream. Secondly, the post-processor increases the entropy per bit of the output stream. This enhancement is achieved through a compression function that distills the entropy from the input stream, producing a slower output stream with heightened randomness.

In addition, the design allows for internal random numbers that can be produced in a more varied way plus the combination of external random number inputs—both increasing the unpredictability of the output. This intricate blend of natural entropy sources, sophisticated algorithmic refinement, and optional post-processing presents superior-quality random numbers. With this level of complexity yet inherent adaptability based on different prerequisites such as those outlined above, these generated random values will be suitable for a broad spectrum of applications.

### 3.1. Classifications of Memristor-Based TRNG

Memristor-based TRNGs have emerged as a promising technology for generating high-quality random numbers, which are crucial for various security applications, such as cryptography and secure communications. These TRNGs exploit the inherent stochastic behavior and intrinsic variability present in memristive devices to introduce randomness into the generated bit sequences. Memristor-based TRNGs leverage properties within memristive devices for randomness, which are classified by entropy sources, mainly including random telegraph noise (RTN), switching behaviors, and delay time [8,10,11,12,14,46,47].

RTN is an inherent phenomenon within memristive devices, characterized by random fluctuations in device conductance or resistance over time. These fluctuations arise from the trapping and detrapping of charge carriers in defect sites within the device material. TRNGs exploit the stochastic nature of RTN to enhance the unpredictability of the generated bit sequences. By meticulously monitoring and amplifying RTN signals, these TRNGs can extract random bits with heightened randomness. Another critical parameter in memristor-based TRNGs is the probability of a memristive device undergoing a resistive state transition, or switching. The inherently stochastic nature of these switching events introduces a significant degree of randomness into the device’s behavior, which can be harnessed for random number generation. By thoroughly analyzing the statistical distribution of switching probabilities, these TRNGs can produce bit sequences with a high level of unpredictability. Furthermore, the delay time, defined as the duration required for a memristive device to transition between different resistive states upon receiving a stimulus, is a pivotal factor. Variations in delay times are influenced by the material properties, device geometry, and operating conditions. Memristor-based TRNGs utilize these variations to introduce randomness into the bit sequences. By accurately measuring and analyzing the distribution of delay times, these TRNGs can derive random bits with substantial entropy.

Table 1 provides an overview of various memristor-based TRNGs, highlighting the sources of randomness, material stacks, endurance, bit generation rates, post-processing requirements, and performance in NIST tests. Each of these memristors employs different mechanisms to harness randomness, presenting unique advantages and limitations.

Different implementations use distinct sources of randomness, such as random telegraph noise (RTN), switching probability, C2C variation, delay time, stochastic oscillation, and thermal fluctuations, to ensure the unpredictability of the output. Common memristors currently used in TRNGs often utilize filamentary switching mechanisms due to the inherent randomness of filament formation and dissolution. Filamentary switching in a memristor involves forming and breaking a conducting filament in a dielectric material, typically consisting of metal ions or oxygen vacancies. The dynamics of conductive filament formation and breakage are highly sensitive to atomic-level variations and thermal fluctuations, resulting in inherently stochastic switching properties. This randomness is crucial for TRNGs because it ensures the unpredictability of the generated random numbers. The bit generation rates of these TRNGs range from as low as 10 b/s to as high as 600 Mb/s, demonstrating significant advancements in the speed and efficiency of random number generation. Post-processing is often applied to improve the quality of the randomness, although some implementations achieve high randomness quality without it. The number of NIST tests passed serves as a benchmark for the reliability and robustness of the TRNGs, with many implementations passing all 15 tests, indicating their suitability for secure applications.

RTN is a prominent source of randomness, attributed to the charge trapping and detrapping at defect sites within the oxide layer of memristors. This process creates discrete current fluctuations that can be harnessed as an entropy source. Key parameters include defect density and energy levels, which influence the amplitude and frequency of RTN. RTN-based TRNGs, such as those utilizing W/TiON/SiO_2_ stacks, are suitable for devices where high-frequency noise is desirable. The Ir/Ta_2_O_5_/TaO_*x*_/TaN memristor [10] stands out with an impressive endurance of $10^{6}$ cycles and a high bit generation rate of $3.2\;\times \;10^{4}$ kb/s, passing all 15 NIST tests, demonstrating its high reliability and robustness. In contrast, the W/TiO_*x*_N_*y*_/SiO_2_ memristor [8] lacks data on bit generation rate and endurance, with poor performance in NIST tests, passing only 5 out of 15. This comparison underscores the superior performance of Ir/Ta_2_O_5_/TaO_*x*_/TaN memristors in leveraging RTN for high-quality TRNG applications. In general, the signal from the NTR is amplified by the sense amplifier and then fed into the associated circuitry to generate the random numbers. This process necessitates the use of a post-processor, which is a common feature of this type of method.

Switching probability is another crucial source of randomness explored in memristors with different material stacks. The probability of switching depends on factors like applied voltage, pulse duration, and material composition. Key considerations include the device’s endurance and the distribution of switching times, which should be broad to maximize randomness. The Cu/AlO_*x*_/TaN memristor [46], with an endurance of $5\times 10^{3}$ cycles, leverages the switching probability for randomness, although data on its bit generation rate are not provided. The Pt/TiO_2−x_/Pt memristor [9] also explores switching probability. This method uses the inherent unpredictability of the switching events in memristors, making it suitable for TRNG applications. The switching frequency typically constrains the bit generation rate of this method. Concurrently, the on–off ratio must be substantial to effectively differentiate between the high and low resistance states, thereby facilitating the generation of random numbers or the incorporation of post-processing procedures to enhance the generation rate.

Cycle-to-cycle variation is utilized as a source of randomness in the Pt/Ta_2_O_5_/Ta memristor [11]. This memristor exhibits an endurance of $2\times 10^{4}$ cycles and a bit generation rate of $1\times 10^{-2}$ kb/s, passing 12 out of 15 NIST tests. The C2C variation method relies on the natural variability in the switching behavior of memristors from one cycle to the next, providing an additional layer of randomness that can be harnessed for TRNG applications. In contrast to the switching probability method, this method is capable of utilizing both high and low resistive states, although it remains constrained by the switching frequency. Furthermore, the memristor must possess a high degree of endurance.

Delay time is another important technique utilized to generate randomness, as seen in Pt/Ag/Ag:SiO_2_/Pt and Ag/SiN_*x*_/n-Si memristors [14,47]. Key parameters include the consistency of the delay time and its sensitivity to environmental variations, which can add to the randomness. The Pt/Ag/Ag:SiO_2_/Pt memristor, with an endurance of $10^{7}$ cycles and a bit generation rate of 6 kb/s, passes all 15 NIST tests, indicating high randomness quality, without the need for post-processing [14]. Conversely, the Ag/SiN_*x*_/n-Si memristor [47], achieves a bit generation rate of $1.12\times 10^{2}$ kb/s and passes all 15 NIST tests, suggesting that it is a promising candidate for TRNG applications despite the absence of endurance data. Furthermore, memristors leveraging delay and relaxation times provide an effective means of generating randomness. These involve the memristor’s response times to voltage changes and its relaxation behavior back to equilibrium. Important parameters include the mean and standard deviation of the delay and relaxation times. The Pt/Cu_0.1_Te_0.9_/HfO_2_/Pt memristor [12], with unspecified endurance and a bit generation rate of $3.2\times 10^{1}$ kb/s, passes all 15 NIST tests, demonstrating reliable performance. Similarly, the Pt/HfO_2_/TiN memristor [48], with an impressive bit generation rate of $10^{5}$ kb/s, passes all NIST tests, highlighting its efficiency in generating high-quality random numbers. Currently, this choice of entropy source is the dominant method to generate random numbers without post-processing, and while endurance leaves much to be desired, this will greatly reduce the complexity and potential power consumption of the system.

Similar to delay time, the stochastic firing pulses also show promising performance. In this mechanism, the inherent delay before a memristor switches under an applied voltage pulse is exploited. Devices like Ag/TiN/HfO_*x*_/HfO_*y*_/HfO_*z*_/Pt memristor [51] exhibit variability in firing times due to material inhomogeneities and defect dynamics. The primary parameter is the statistical distribution of delay times, which should be wide to enhance entropy. The Ag/TiN/HfO_*x*_/HfO_*y*_/HfO_*z*_/Pt memristor achieves an endurance of $10^{6}$ cycles and a bit generation rate of $1.08\times 10^{2}$ kb/s, excelling in randomness quality by passing all 15 NIST tests. This demonstrates the effectiveness of stochastic firing pulses in generating high-quality random numbers, making this memristor highly suitable for secure applications.

Stochastic oscillation is a significant entropy source in memristor-based TRNGs, where the oscillation frequency and amplitude are inherently random due to thermal noise and other intrinsic fluctuations. In devices like volatile memristors paired with resistors or capacitors, the oscillation can be highly sensitive to initial conditions and environmental factors. Key parameters for stochastic oscillation include the quality factor of the oscillator, the thermal noise level, and the intrinsic variability of the memristor’s switching characteristics. Higher quality factors can lead to more stable oscillations, while higher thermal noise levels can enhance entropy but might also introduce stability concerns. The Pt/Ti/NbO_*x*_/Pt memristor [49] exhibits an impressive endurance of $4\times 10^{8}$ cycles and a bit generation rate of $4\times 10^{1}$ kb/s, successfully passing all 15 NIST tests. Additional implementations include the W/GeTe_*x*_/W and Pt/LaCoO_3_/Pt/LaAlO_3_ memristors [13,50], with endurance values of $2\times 10^{9}$ and $6\times 10^{8}$ cycles, respectively. Both devices achieve high bit generation rates and pass all NIST tests, thereby demonstrating the robustness of stochastic oscillation in TRNGs. The W/GeTe_*x*_/W memristor, with a bit generation rate of $2.22\times 10^{3}$ kb/s, and the Pt/LaCoO_3_/Pt/LaAlO_3_ memristor, with an impressive bit generation rate of $6\times 10^{5}$ kb/s, both demonstrate their efficiency in generating high-quality random numbers. Stochastic oscillation is an effective method for generating randomness in memristors, offering high endurance, impressive bit generation rates, and robust performance in NIST tests.

The choice of entropy source depends on the specific characteristics and application requirements of the memristor-based TRNG. For high-speed applications, switching probability and delay times are advantageous due to their rapid response. For low power, delay time is preferred due to its lack of post-processing and high bit generation rate. Stochastic oscillation is particularly useful in scenarios where continuous and high-quality randomness is required, provided that the device can manage the associated thermal and stability challenges. By analyzing different sources of randomness and their implementations, it is evident that memristor-based TRNGs exhibit diverse capabilities and efficiencies. Their performance in terms of bit generation rates, endurance, and NIST test results demonstrates the potential for secure and reliable random number generation across various applications.

**Figure 3:**
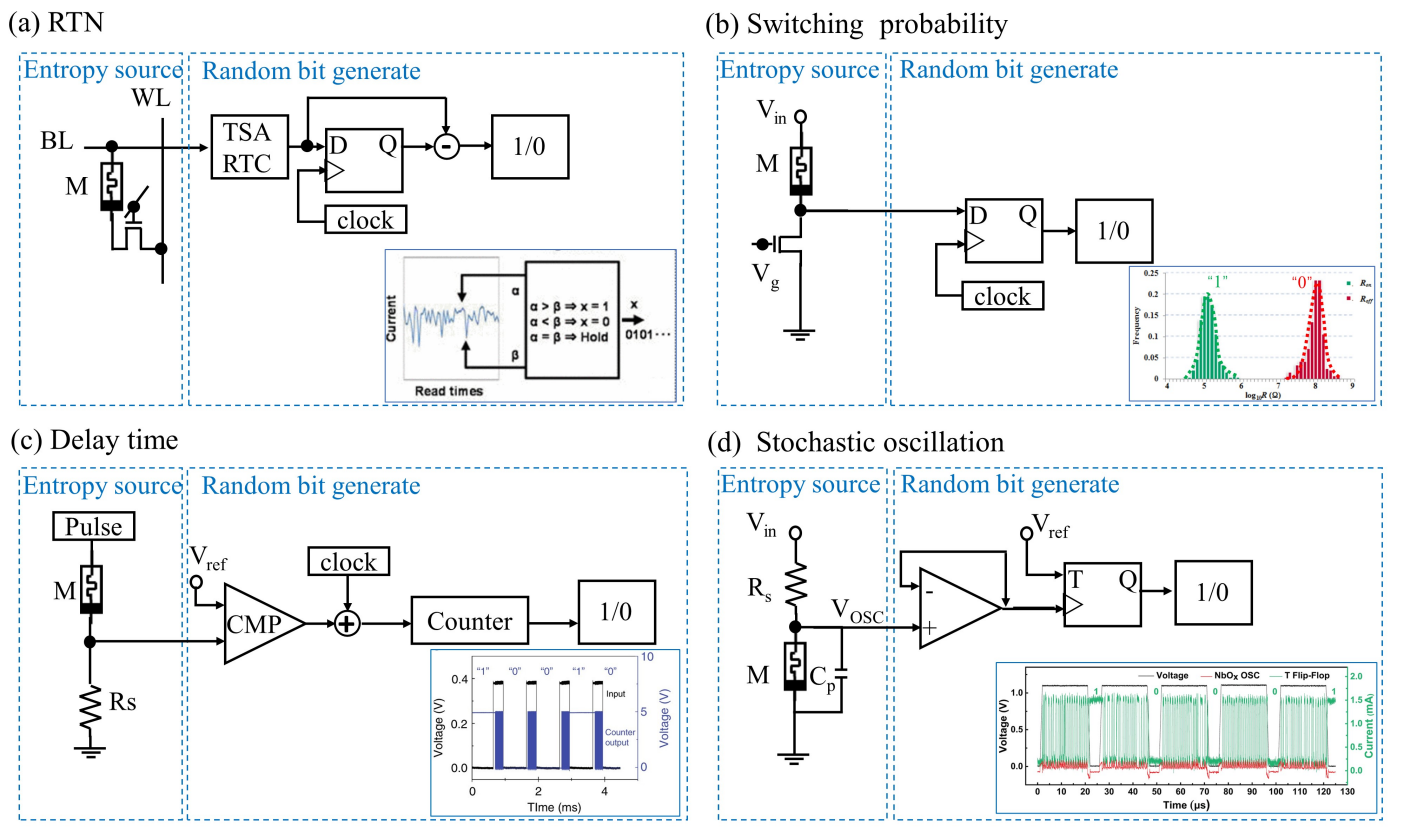
Circuit schematic of a memristor-based TRNG using various entropy sources. (**a**) RTN: The current fluctuations within the memristor are sampled and processed using a timing sense amplifier (TSA) and a resistance-to-time converter (RTC), followed by post-processing to generate random bits. Inset illustrates the comparison of current differences at various read times. Adapted from Ref. [10]. (**b**) Switching probability: The dynamic switching of the memristor between high and low resistance states, controlled by programming voltages and durations, generates random bits for capture and processing by the circuitry. The inset shows the statistical distribution of the high resistance state (Roff) and low resistance state (Ron), representing binary 0 and 1, respectively. Adapted from Ref. [9]. (**c**) Delay time: This method utilizes the delay time before a memristor switches ON under the influence of a voltage pulse as the entropy source. This delay is measured using a comparator, which subsequently generates a digital output. The inset displays the timing behavior of the output in relation to the input pulses. Adapted from Ref. [14]. (**d**) Stochastic oscillation: Thermal fluctuations in a volatile memristor connected in series with a resistor act as the entropy source. The oscillations produced are processed by a comparator and a T flip-flop. The inset demonstrates six consecutive cycles of random bit generation. Adapted from Ref. [49].

Figure 3 presents various circuit schematics of a memristor-based TRNG using various entropy sources and random bit generate circuits, which are crucial for generating unpredictable random numbers. Figure 3a illustrates a TRNG circuit where the entropy source is the random telegraph noise in a memristor. The fluctuating current from the memristor is read via bit line (BL) and word line (WL), sampled by a time-to-digital converter (TSA) and resistance-to-time converter (RTC). These samples are then processed by a D flip-flop, clocked by an external clock, and undergo further post-processing to generate true random numbers. The inset shows current differences at different read times, demonstrating the effectiveness of noise as a source of entropy. In Figure 3b, the operation of the 1-branch memristor-based TRNG involves the memristor dynamically switching between high and low resistance states based on programming voltages and durations, leading to the generation of random bits. The memristor’s stochastic switching generates random bits, which are then captured and processed by the circuit for further use. The 1-branch TRNG design can operate over a wide frequency range, from Hz to GHz, allowing for high-speed random number generation. By adjusting the programming voltages and durations of the memristor, the 1-branch MTRNG can adapt to different operating conditions. Figure 3c depicts a TRNG design where the entropy source is the intrinsic delay time before a memristor switches ON under a voltage pulse. The random delay time of the memristor causes the pulse width after passing through the comparator to vary, and when combined with the clock signal, the number of clock pulses sent to the counter will also vary. Because the delay time is random, the number of clock pulses sent to the counter is also random, and the final output of the counter will be random. The inset displays the timing behavior of the output in response to input pulses, highlighting the stochastic nature of the switching delay. Figure 3d presents an oscillator circuit involving a volatile memristor paired with a series resistor. The entropy source here is the thermal fluctuations during the oscillation of the NbO_*x*_-based threshold switching (TS) device, which includes a niobium-oxide-based memristor and an internal capacitor. NbO_*x*_ TS devices can exhibit oscillatory current behavior. During oscillation, the capacitive elements in the device affect the oscillation frequency by inducing a temperature-dependent RC delay. As a result, the heat generation and dissipation conditions are random, resulting in slight variations in the oscillation period. These fluctuations contribute to the device’s oscillatory behavior, which is essential for TRNG applications.

The four examples in Figure 3 together highlight the diversity and effectiveness of varying entropy sources in memristor-based TRNGs. Each design exploits unique physical phenomena (RTN, switching probability, delay time, and stochastic oscillation) to generate truly random numbers. These methods ensure robustness and unpredictability, critical for secure communications and cryptographic systems applications. At the same time, the origin of the different entropy sources also determines the complexity of the circuit. The use of memristors as core components in these TRNGs highlights their potential for low-power, high-density random number generation, which is critical for modern electronics and security applications. However, the impact of the number of devices other than memristors on the performance and power consumption of the entire system also needs to be considered. In summary, it is beneficial to reduce the complexity of the circuit as much as possible, especially the reduction in the complexity of post-processing or the absence of post-processing parts to reduce the power consumption of the entire system.

Memristor-based TRNGs leverage the inherent stochastic properties of memristive devices. By exploiting phenomena such as RTN, switching behavior, and delay time, these TRNGs produce random bit sequences with high entropy, ensuring their suitability for advanced security applications. We have outlined the various methods and material compositions used in memristor-based TRNGs [9,10,14,49]. Each of the methods utilized in a memristor-based TRNG has its unique advantages and disadvantages. Memristors using RTN have high endurance and bit generation rate [10], but usually require extensive post-processing to ensure reliability. In contrast, those using delay times generally have good endurance, but the bit generation rate is generally not as high as RTN [12,14,47], and this method does not require post-processing. Switching probability-based TRNG benefits from the most basic switching characteristics of memristors, and the circuit implementation is simple, which is conducive to reducing power consumption [9]. This comprehensive comparison highlights the versatility of memristor-based TRNGs and the continued progress in optimizing their performance for safe and reliable random number generation in a variety of applications.

### 3.2. Comparison with CMOS Technologies

In the realm of number generation, the introduction of memristor-based TRNGs has sparked significant curiosity. These new circuits are anticipated to provide simplicity, compact size, and durability when compared to TRNGs relying on complementary metal–oxide semiconductor (CMOS) technologies.

One significant advantage of memristor-based TRNGs lies in their simplified circuitry. Almost all traditional TRNGs are based on random noise in CMOS digital and analog circuits, such as thermal and shot noise [52,53,54,55,56]. These CMOS-based TRNGs often require multistage voltage amplifiers to extract randomness from MOSFET thermal noise, resulting in complex circuit designs. In contrast, memristor-based TRNGs leverage the inherent stochasticity of memristive devices, eliminating the need for amplifiers and streamlining circuit design [9,14]. For example, a typical CMOS-based TRNG might require a complicated chain of inverters and amplifiers to generate sufficient noise, while a memristor-based TRNG can achieve similar results with a single memristive device and minimal supporting circuitry. This simplification not only reduces the chip area required but also potentially improves the overall reliability of the system by reducing the number of components that could fail.

In terms of speed, memristors can switch states in nanoseconds, comparable to high-speed CMOS circuits. However, memristors have the added advantage of nonvolatility, which reduces power consumption by eliminating the need for constant refreshing. Furthermore, memristor-based TRNGs offer compact circuit layouts and reduced power consumption. Research indicates that these circuits can achieve a bit generation rate of 2.22 Mb/s while consuming as little as 717.1 pJ/bit, as reported in reference [50]. This efficiency is attributed to the high oscillation speed and high endurance of the device, with each single oscillation capable of generating one random bit. This reduces the number of operations required to generate a random bit, thereby minimizing energy consumption during random number generation. In contrast, CMOS-based TRNGs often require continuous power to maintain their noise-generating circuits, which results in higher overall power consumption.

Both CMOS-based and memristor-based TRNGs face their unique challenges. CMOS-based TRNGs often suffer from random noise being masked by deterministic interference due to their lower amplitude, and their raw outputs often exhibit large “1”/“0” deviations. In addition, they require complex preamplifier circuits to amplify smaller noise amplitudes, which inevitably increases power consumption. Memristor-based TRNGs face challenges in achieving high bit generation rates. Early memristor-based TRNGs exhibited lower rates, around 6 kbs^−1^ [14], but currently reach up to the Mbs^−1^ range [50]. This is because memristor-based TRNGs are considerably slower than mainstream CMOS technology, which operates at speeds up to GHz. This difference is attributed to the micrometer size of the memristor, in contrast to the nanometer size of CMOS technology. As expected, reducing the size of our prototype components should significantly increase speed without introducing a fundamental bottleneck. The inclusion of a post-processing step has the potential to improve the bit generation rate.

Another consideration is post-processing complexity. Bit streams generated by both CMOS and memristor-based TRNGs often require post-processing before undergoing rigorous testing, such as the NIST test. While CMOS-based TRNGs may require simpler post-processing, the specific characteristics of memristive devices often necessitate more extensive correction. For instance, a study by Zhang et al. [11] found that raw bit streams from their memristor-based TRNGs required a complexity post-processing algorithm to pass the NIST tests. One of the considerations in the design of memristor-based TRNGs is the reliability of the system. The reliability of a memristor-based TRNG is contingent upon the endurance of the memristor itself. For example, a study by Fu et al. [50] demonstrated that their GeTe_*x*_ memristor-based TRNG exhibited consistent performance over 10^9^ switching cycles. However, beyond this point, the endurance quality is observed to degrade [57], which affects the reliability of the memristor-based TRNG. In contrast, CMOS-based TRNGs rely on the fundamental physical characteristics of the transistor and can operate reliably for much longer periods without significant performance degradation.

Hardware cost is another area where memristors excel. The simpler architecture of memristors, often requiring fewer transistors and components than CMOS circuits, leads to reduced manufacturing costs. Additionally, the ability to integrate memristors onto existing CMOS platforms can further lower costs by leveraging existing fabrication infrastructure. Scalability is a critical factor for future technology nodes. Memristors can be scaled down to atomic dimensions, offering higher density and integration levels than CMOS. This scalability advantage is crucial for applications requiring large arrays of TRNGs, such as in cryptographic systems and secure communications.

In conclusion, memristor-based TRNGs have the potential to enhance the generation of randomness, while offering advantages in terms of circuit simplicity, power efficiency, and miniaturization compared to conventional CMOS-based designs. Nevertheless, there are still obstacles to overcome to achieve high bit generation rates, to manage the complexity of post-processing, and to ensure long-term reliability. It is imperative to address these challenges to fully utilize the capabilities of memristor-based TRNGs and advance their integration into a variety of applications, including cryptography and secure electronics. Potential future research directions may include exploring new memristor materials with improved switching speeds and durability, developing more efficient post-processing algorithms for memristor-based TRNGs, and investigating hybrid designs that combine the advantages of memristor and CMOS technologies. This will create more robust and versatile TRNG solutions.

## 4. Case Study: Entropy Source Based on Electroforming Free YMO Memristor for TRNG

Resistive switching devices, particularly memristors, are gaining significant attention for their potential in nonvolatile memory applications. Among these, yttrium manganite (YMnO_3_, YMO) thin films present a compelling case due to their unique electroforming-free digital resistive switching behavior [28]. In particular, YMO memristors exhibit robust endurance, with the number of load cycles improved to the order of $10^{3}$ in specific compositions with Au top and Pt bottom electrodes. This improvement is essential for practical applications where device lifetime is a key consideration. The on/off ratio, which indicates the contrast between HRS and LRS, is another critical parameter. The retention of these memristors is exceptional, with the memory window characterized by an on/off ratio ranging from $10^{4}$ to $10^{6}$, depending on the electrode materials, sizes, and YMO compositions [28]. Such a large memory window ensures a clear distinction between HRS and LRS, which is critical for reliable data storage and retrieval. The underlying mechanism for resistive switching in YMO memristors involves the formation and breakage of conductive filaments within the insulating matrix, a process driven by thermochemical phenomena. These filaments exhibit stability that results in excellent retention properties.

Our case study focuses on the critical parameters of on/off ratio, C2C variation, resistive switching behavior, and switching frequency in YMO memristors that are critical to their performance in TRNG applications. We contend that electroforming and digital YMO devices with stochastic behavior are well suited for the construction of TRNG. These devices provide the necessary unpredictability and robust statistical properties required for generating high-quality entropy sources for TRNG.

**Figure 4:**
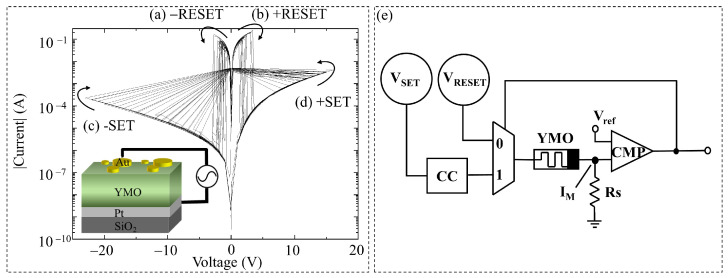
I–V characteristic of a single YMO memristive cell with 100 cycles in (**a**) −RESET, (**b**) +RESET, (**c**) –SET, (**d**) +SET processes. TE size is 0.453 mm^2^. Schematic illustration of room-temperature DC electrical measurement setup for a YMO-based resistive switch connected in series with a DC source meter. (**e**) Circuit schematic of YMO memristor-based TRNG. The YMO memristive cell is controlled by two source voltage ($V_{SET}$/$V_{RESET}$). The random voltage value over resistance (Rs), and the set bias (or reset bias) are compared with the set (or reset) reference voltage by DPDT relay.

Figure 4 depicts the I–V characteristics of a single YMO memristive cell undergoing 100 cycles under positive and negative bias ranges. The structure of the YMO memristor and a schematic diagram of the measurement setup in series with a DC source meter are depicted in the insets. The I–V curves display the resistive switching behavior of YMO memristors, consistently demonstrating unipolar resistive switching. The figure illustrates four distinct processes within the memristor: reset processes in Figure 4a,b, and set processes in Figure 4c,d. During the set process, the voltage gradually rises alongside a corresponding increase in current. Upon exceeding the threshold voltage, the device switches from HRS to LRS by forming conductive filaments. On the other hand, in the reset process, the device reverts to HRS above the threshold voltage by disrupting the conductive filaments. It is noteworthy that the set process requires higher voltage and lower current, while the reset process, transitioning from LRS to HRS, demands lower voltage and higher current. Set transitions typically occur within a bias range of ±5 V to ±30 V, while reset transitions take place within a bias range of ±0.5 V to ±4.0 V. In both scenarios, the switching is sudden and remains unaffected by bias polarity. The depiction of multiple cycles of a single YMO memristor in Figure 4 reveals varying voltage values during set and reset processes, showcasing significant periodic variations that confirm the stochastic nature of their switching behavior. According to the promising stochastic distribution of SET and RESET biases in YMO memristors, we propose to use YMO memristors as entropy sources in TRNG with the potential of high randomness for security-oriented applications.

The basic principle of a YMO memristor-based TRNG is introduced, and the circuit design of YMO memristor-based TRNG was built. The general idea of a YMO memristor-based TRNG is the following. The random set or reset biases are sampled periodically and compared to a certain predefined threshold voltage. The threshold voltage is derived from the mean value of the Gaussian distribution of the set or reset biases. If the sampled set or reset biases is higher than this predefined mean threshold of the set or reset biases, it is assigned a value of “1”; otherwise, it is assigned “0”.

**Figure 5:**
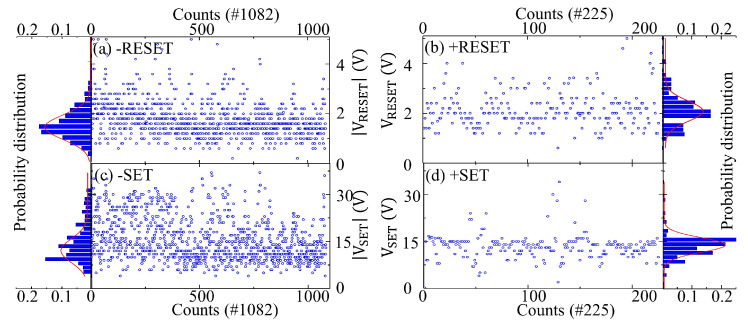
The statistics of the set or reset voltage values from the first cycle to the last cycle, (**a**) in negative RESET with 1082 load cycles, (**b**) in positive RESET with 225 load cycles, (**c**) in negative SET with 1082 load cycles, and (**d**) in positive SET with 225 load cycles. The probability distribution histogram of the bias in the four different processes of a single YMO memristive cell is depicted in the insets.

The circuit schematic of a YMO memristor-based TRNG is depicted in Figure 4e. It comprises a YMO memristive cell, SET/RESET source voltages, a DPDT (double pole double throw) relay, and a comparator. The YMO memristive cell is regulated by $V_{SET}$/$V_{RESET}$ source voltages, which provide a sweeping bias to induce the continuous generation of set bias or reset bias. The random voltage value over resistance (Rs) is linked in series with the YMO memristive cell. Subsequently, the set bias or reset bias is compared with the set or reset reference voltage to yield a binary 0 or 1. The resulting binary numbers are then tallied by a counter to create a random bit stream. During the YMO switching process, both SET and RESET occur within a specific range, allowing each SET or RESET process to yield random bits. The reference voltage $V_{ref}$ remains constant, with both SET reference voltage ($V_{ref,SET}$) and RESET reference voltage ($V_{ref,RESET}$) defined by the mean of a Gaussian distribution, as illustrated in Figure 5.

Given the unipolar nature of the YMO memristor resistive switching, a reset is required after each set operation before setting it again. To achieve this, a DPDT relay is employed to alternate between SET and RESET operations. This design offers the advantage of continuously outputting random numbers generated by the memristor’s SET and RESET processes concurrently. The resulting random number stream alternates between SET and RESET, enhancing both the efficiency and randomness of the output bits in the proposed YMO-based TRNG.

The statistical test results of YMO memristor-based TRNG are demonstrated for the YMO memristor-based TRNG under SET/RESET biases with a pulse width of 100 ms and for the YMO memristor-based TRNG under SET/RESET biases with different pulse widths. The purpose of the YMO memristor-based TRNG with different pulse widths is given as a potential feature for further application in stochastic computing. Statistical results of YMO memristor-based TRNG with source pulse width 100 ms are given. According to the proposed design for YMO memristor-based TRNG in Figure 4e, 1082 cycles in the negative bias range and 225 cycles in the positive bias range are recorded. After large load cycle measurements, the statistical results of a single memristive cell are analyzed.

The statistics of the set or reset voltage values from the initial to the final cycle can be derived from Figure 5. Notably, the negative bias range exhibits superior endurance, with 1082 load cycles at maximum in the negative bias range compared to 225 in the positive bias range. Figure 5 illustrates that the distribution of negative reset biases is relatively random, primarily falling between −0.5 V and −4 V. Similarly, in Figure 5b, the distribution of positive reset biases is random, concentrated mainly between 1 V and 4 V. In Figure 5c,d, the distributions of negative and positive set biases, respectively, show a similar random pattern, with concentrations between −5 V and −35 V and 8 V and 16 V. By analyzing the cyclic bias statistics, one can generate a probability distribution map of the bias.

As depicted in Figure 5, the histogram illustrates the probability distribution of bias in four distinct processes of a single YMO memristive cell. Figure 5a displays the probability distribution of reset biases in the negative bias range after 1082 load cycles. The histogram reveals a Gaussian distribution pattern for the reset biases. Similarly, Figure 5b showcases the probability distribution of reset biases in the positive bias range following 225 load cycles, exhibiting a Gaussian distribution. Moving on to Figure 5c, it illustrates the probability distribution of set biases in the negative bias range with 1082 load cycles, displaying a Gaussian distribution. Lastly, Figure 5d presents the probability distribution of set biases in the positive bias range after 225 load cycles, showing a Gaussian distribution pattern as well.

The analysis focused on the statistical outcomes of a single YMO memristive cell under varying pulse widths. Specifically, four different pulse widths (100 ms, 200 ms, 500 ms, 1000 ms) were applied to the same YMO memristive cell, with each width subjected to 100 load cycles. Due to the superior endurance exhibited in the negative bias range and the limited load cycles in the positive bias range, the statistical evaluation was confined to the negative bias range for this single YMO memristive cell across different pulse widths.

**Figure 6:**
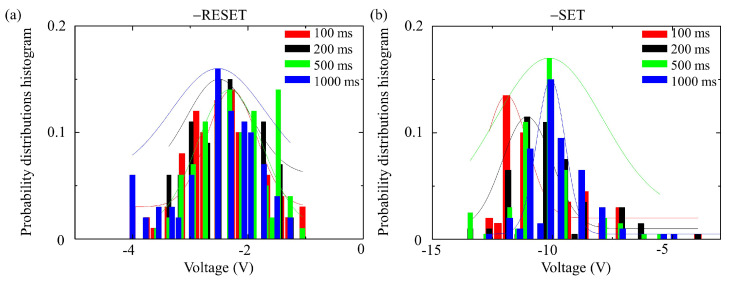
A single YMO memristive cell with pulse widths of 100 ms, 200 ms, 500 ms, 1000 ms, (**a**) −RESET with 100 load cycles and (**b**) −SET with 100 load cycles.

As depicted in Figure 6, the probability distribution histogram illustrates a single YMO memristive cell with pulse widths of 100 ms, 200 ms, 500 ms, and 1000 ms within the negative bias range. Figure 6a represents the probability distribution of reset biases in the negative bias range. All four distributions for different pulse widths exhibit a Gaussian distribution. The Gaussian distribution remains relatively stable despite changes in pulse width. Figure 6b displays the probability distribution of set biases in the negative bias range. Similarly, all distributions for the four pulse widths conform to a Gaussian distribution. However, with variations in pulse width, the Gaussian distribution noticeably shifts. With an increase in pulse width, the corresponding Gaussian distribution shifts to the right, indicating a movement toward the higher bias range.

The stochastic distribution of switching biases in YMO memristive cells during SET and RESET processes makes them an ideal source of randomness for TRNG designs. Given the random and unpredictable nature of the switching bias in the YMO memristive cell during the SET and RESET processes, the YMO memristor is proposed as a potential random source for the TRNG design. The following three aspects are used to evaluate the random performance of the three aforementioned aspects:

1. The periodicity of the phenomenon under investigation. The pseudorandom number generator (PRNG) is calculated by a mathematical formula with periodicity, which itself must also exhibit periodicity. This is defined as a sub-sequence in the sequence being identical to another sub-sequence. The period must be sufficiently long to provide the requisite data for the intended application. A sequence of true random numbers is the only means of obtaining a true, never-repetitive sequence of random numbers. The YMO memristor-based TRNG is determined by the physical intrinsic stochastic behavior of the memristor itself. As illustrated in Figure 5, negative SET/RESET biases employed to generate the random number exhibit random behavior within the range of 1 to 1082, and there is no discernible periodic change. Furthermore, the applied biases satisfy the Gaussian distribution.

2. Correlation. It is necessary that the random numbers in a random number sequence generated by a random number generator be uncorrelated, and that the random numbers in the generated random number sequences also be uncorrelated. In the case of a PRNG, the mathematical formulas used must be carefully designed to meet the unrelated requirements as much as possible. The sequence of true random numbers naturally satisfies this irrelevance.

3. Distribution uniformity. The sequence of random numbers obeys a uniform distribution, that is, the probability of occurrence of any number in the same range is the same. Here, uniformity refers to the variability observed in the current levels during the set and reset processes. This variability can lead to inconsistencies in the memristive states, affecting the reliability of the random bits generated. However, due to the poor endurance of the YMO memristor, it is impossible to generate enough data, and the current uniformity is not optimal.

In conclusion, this section presents the statistical results of the YMO memristor-based TRNG circuit design. Furthermore, the random performance of TRNG is analyzed based on the experimental results. Moreover, memristors based on TRNG are capable of providing satisfactory randomness properties.

## 5. Challenge and Outlook

The development and deployment of TRNGs utilizing memristive technologies presents a multitude of challenges and opportunities. One significant challenge is the inherent variability and stability of memristor devices. The reliance on filamentary switching mechanisms, while advantageous for generating randomness due to their stochastic nature, also leads to challenges in consistency and reproducibility. Variations in filament formation and rupture can impact the reliability and lifespan of the devices, posing significant hurdles for widespread adoption in commercial applications. Another critical issue is the scalability of memristor-based TRNGs. As the demand for higher bit generation rates increases, ensuring that memristor arrays can scale while maintaining their stochastic properties becomes essential. The integration of these devices with existing semiconductor technologies without compromising their performance or introducing significant complexity remains a substantial challenge.

Despite these challenges, the outlook for memristor-based TRNGs is promising. Advances in material science and nanofabrication techniques continue to enhance the performance and reliability of memristive devices. Optimizations in the material stack and switching mechanisms, as observed with various configurations like Pt/Ti/NbO_*x*_/Pt [49] and W/GeTe_*x*_/W [50], offer pathways to improve endurance and bit generation rates. Additionally, the development of hybrid systems that combine the advantages of different types of memristors, such as integrating diffusive and filamentary memristors, could provide more robust solutions. Innovative circuit designs and algorithms for entropy extraction and post-processing also hold the potential to address some of the current limitations. By refining these methods, it is possible to achieve higher throughput and better compliance with randomness standards without significantly increasing the system’s complexity or power consumption. Moreover, future research should focus on combining memristive TRNG with existing semiconductor technologies, exploring new materials and device architectures, and developing strong test standards to verify its reliability and security in practical applications.

## 6. Conclusions

This paper has presented a comprehensive exploration of memristor-based TRNGs, highlighting their intrinsic potential for generating high-quality random numbers, which are essential for cryptographic systems and security protocols. Our investigation underscores the unique advantages of memristor-based TRNGs, including their high endurance, robust statistical properties, and the ability to generate random numbers with high entropy without extensive post-processing. The comparative analysis with CMOS-based TRNGs reveals that memristive devices offer a promising alternative, with simplified circuitry and reduced power consumption. Furthermore, the case study of the electroforming-free YMO memristor as an entropy source exemplifies the practical application of memristive technology in TRNGs, showcasing its effectiveness in producing secure and unpredictable random numbers.

The YMO memristor, with its electroforming-free and unipolar resistive switching characteristics, is pivotal for sustainable entropy generation. The Gaussian distribution of SET and RESET biases, revealed through rigorous cycling tests, is a testament to the device’s intrinsic randomness, making it an ideal candidate for TRNGs. The innovative circuit design proposed herein effectively captures the stochastic nature of YMO memristors, enabling the generation of random bits that are both secure and efficient.

Current research on YMO memristor-based TRNGs faces key challenges such as achieving high bit generation rates and ensuring long-term reliability. While acknowledging that further research is necessary to improve post-processing algorithms and fully realize the reliability and longevity of memristor-based TRNGs, the results presented in this paper provide a strong argument for integrating YMO memristors into next-generation security applications. Future work will focus on optimizing the post-processing complexity of TRNGs and improving their performance to meet the dynamic needs of cryptographic systems and other security-sensitive applications.

As we look to the future, the memristor-based TRNGs present a fertile ground for further research and development. The pursuit of optimizing memristor materials, enhancing switching speeds, and ensuring long-term reliability are key areas that require attention. In conclusion, integrating memristive devices into security applications represents a significant advancement, offering a future where hardware security benefits from the inherent unpredictability of memristor technology. This fusion promises enhanced efficiency and robust protection in security systems.

## Figures and Tables

**Table 1:** Overview of different memristor-based TRNGs with different sources of randomness.

Source of Randomness	Material Stack	Endurance (Cycle #)	Bit Generation Rate (kb/s)	Post-Processing	NIST Tests (n/15)	Year References
RTN	W/TiO_*x*_N_*y*_/SiO_2_	N/A	N/A	Yes	5/15	2012 [8]
	Ir/Ta_2_O_5_/TaO_*x*_/TaN	$10^{6}$	$3.2\times 10^{4}$	Yes	15/15	2016 [10]
Switching probability	Cu/AlO_*x*_/TaN	$5\times 10^{3}$	N/A	Yes	N/A	2015 [46]
	Pt/TiO_2_/Ti$O_{2-x}$/Pt	N/A	N/A	Yes	N/A	2015 [9]
Cycle to cycle variation	Pt/TaO_*x*_/Ta	$2\times 10^{4}$	$1\times 10^{-2}$	Yes	12/15	2017 [11]
Delay time	Pt/Ag/Ag:SiO_2_/Pt	$10^{7}$	6	No	15/15	2017 [14]
	Ag/SiN_*x*_/n-Si	N/A	$1.12\times 10^{2}$	No	15/15	2024 [47]
Delay and relaxation times	Pt/Cu_0.1_Te_0.9_/HfO_2_/Pt	$4.8\times 10^{7}$	$3.2\times 10^{1}$	No	15/15	2021 [12]
	Pt/HfO_2_/TiN	N/A	$10^{5}$	No	15/15	2020 [48]
Stochastic oscillation	Pt/Ti/NbO_*x*_/Pt	$4\times 10^{8}$	$4\times 10^{1}$	No	15/15	2021 [49]
	W/GeTe_*x*_/W	$2\times 10^{9}$	$2.22\times 10^{3}$	Yes	12/15	2023 [50]
	Pt/LaCoO_3_/Pt/LaAlO_3_	$6\times 10^{8}$	$6\times 10^{5}$	No	15/15	2024 [13]
Stochastic firing pulses	Ag/TiN/HfO_*x*_/HfO*_y_*/HfO_*x*_/Pt	$10^{6}$	$1.08\times 10^{2}$	No	15/15	2022 [51]

## Data Availability

Data will be made available upon request.
